# Induction of a 5-lipoxygenase product by daidzein is involved in the regulation of influenza virus replication

**DOI:** 10.3164/jcbn.19-70

**Published:** 2020-01-01

**Authors:** Yuka Horio, Riho Sogabe, Mototada Shichiri, Noriko Ishida, Ryosuke Morimoto, Atsushi Ohshima, Yuji Isegawa

**Affiliations:** 1 Department of Food Sciences and Nutrition, Mukogawa Women’s University, 6-46 Ikebiraki, Nishinomiya, Hyogo 663-8558, Japan; 2 Biomedical Research Institute, National Institute of Advanced Industrial Science and Technology (AIST), 1-8-31 Midorigaoka, Ikeda, Osaka 563-8577, Japan; 3 DBT-AIST International Laboratory for Advanced Biomedicine (DAILAB), 1-1-1 Higashi, Tsukuba-shi, Ibaraki 305-8562, Japan; 4 Genomics Program, Nagahama Institute of Bio-Science and Technology, 1266 Tamura-Cho, Nagahama, Shiga 526-0829, Japan; 5 Institute for Biosciences, Mukogawa Women’s University, 6-46 Ikebiraki, Nishinomiya, Hyogo 663-8558, Japan

**Keywords:** influenza virus, daidzein, lipid peroxide, signal transduction, lipoxygenase

## Abstract

This study was conducted to evaluate the regulation mechanism of influenza virus replication following treatment of Madin-Darby canine kidney cells with the soy isoflavone daidzein. We performed comparative qualitative and quantitative analyses of lipid peroxide between mock-infected and virus-infected cells treated with or without daidzein, as it had been reported that daidzein was an antioxidant and lipid peroxide levels increased upon virus infection. Contrary to our belief, lipid peroxides were not elevated in virus-infected cells and no decrease in lipid peroxides was observed in daidzein-treated cells. In daidzein-treated cells, 5-hydroxyeicosatetraenoic acid, the 5-lipoxygenase product derived from arachidonate, was significantly elevated compared to other lipid peroxides. Zileuton (5-lipoxygenase inhibitor) and 5-lipoxygenase knockdown reduced the daidzein-induced antiviral effect. Moreover, virus replication was regulated by treatment with 5-hydroperoxyeicosatetraenoic acid, a precursor of 5-hydroxyeicosatetraenoic acid and 5-lipoxygenase primary product. These results suggest that daidzein regulates virus replication via signal transduction through 5-lipoxygenase products.

## Introduction

Influenza viruses, particularly A viruses, cause epidemics and pandemics in human populations, resulting in enormous suffering and economic losses.^(1)^ Influenza virus infections can cause severe complications such as pneumonia and encephalitis and can increase the rate of hospitalization and mortality, particularly in young children and elderly people.^(2,3)^ The M2 ion channel inhibitors amantadine and rimantadine and neuraminidase inhibitors zanamivir and oseltamivir have been used to treat influenza virus infections. Recently, neuraminidase inhibitor-resistant influenza A viruses were reported and an antiviral reagent against RNA-dependent RNA polymerase in influenza virus was developed.^(4–7)^

We previously reported that soybean extract exerted antiviral activity against influenza virus growth.^(8)^ During influenza infection, reactive oxygen species (ROS) and lipid peroxides are generated in several tissues such as the lung.^(9–13)^ Soy isoflavones, such as daidzein, glycitein, and genistein,^(14)^ are a group of compounds present in soybean and function as potent antioxidants.^(15,16)^ Jin *et al.*^(17)^ reported the effect of soy isoflavones, including daidzein (61.7%), glycitein (21.5%) and genistein (16.2%), on diabetic rats. They showed that an improvement in postprandial blood glucose levels and a significant suppression of blood lipid peroxide can be achieved by single or long-term administration of soy isoflavones, respectively.^(17)^ Additionally, soy isoflavones act as phytoestrogens and interact with animal and human estrogen receptors and produce non-hormonal effects.^(18–24)^ We found that daidzein and glycitein inhibited influenza virus replication and suggested that the regulation by daidzein is not directly associated with viral enzymes but rather with host-cellular metabolism (unpublished data).

In this study, we first confirmed whether daidzein exerts antiviral activity through its antioxidant function by measuring lipid peroxide levels. Furthermore, we identified a lipid oxidation product specifically increased in daidzein-treated cells as a lipid mediator that induces antiviral activity and analyzed its production mechanism.

## Materials and Methods

### Cells and viruses

Madin-Darby canine kidney (MDCK) cells were grown in Eagle’s minimum essential medium (MEM; Sigma-Aldrich, St. Louis, MO) containing 70 ml/L fetal bovine serum (FBS). The influenza A virus H1N1 (PR/8/34) was used throughout the experiments. For cell infection, the virus was diluted in serum-free MEM supplemented with 0.4 g/L bovine serum albumin (Sigma-Aldrich), which was adsorbed to cells at a multiplicity of infection (MOI) of 0.001 for 1 h at 37°C. The inoculum then was removed and replaced with FBS-free Dulbecco’s Modified Eagle Medium (DMEM) supplemented with 4 g/L bovine serum albumin and acetyltrypsin (2 µg/mL; Sigma-Aldrich) for the remainder of the infection period.

### Focus forming reduction assay for virucidal activity

Sample influenza viruses used in the virucidal activity assay were prepared by infecting monolayer sheets of MDCK cells with viruses (100 µl, MOI 0.001) in 24-well plates for 1 h as described above. The inoculum was removed, washed once with serum-free MEM, and FBS-free DMEM supplemented with daidzein was added (Kanto Chemical Co., Inc., Tokyo, Japan). After 24 h, the supernatants were harvested and used as the influenza virus samples for focus forming reduction assay (FFRA).

Focus formation was induced as described by Nagai *et al.*^(8)^ Each virus dilution was selected to give a final count of approximately 30 focus-forming units (FFU) per well. Virucidal activity was expressed as the reciprocal of the highest dilution that reduced the number of foci to ≤50% of the control value.

### Sample preparation for lipid peroxide

Virus-infected cells for analysis of lipid peroxidation products were prepared by infecting monolayer sheets of MDCK cells (1 ml, MOI 0.001) in 10-cm dishes for 1 h as described above. The inoculum then was removed and washed once with serum-free MEM, after which FBS-free DMEM supplemented with or without 275 µM daidzein was added. After 24 h, the dishes were washed twice with phosphate-buffered saline (PBS). The cells were harvested into 1.5-ml tubes containing 500 µl of ice-cold PBS using a cell-scraper. The samples were placed on ice immediately after collection. The cells were obtained by centrifugation at 12,500 × *g* for 5 min at 4°C, the supernatant was discarded, and 140 µl of ice-cold PBS was added to the tube. The cells were homogenized with a sonicator. The homogenate (110 µl) was transferred to a new tube, and then 4 volumes of methanol containing 100 µM 2,6-di-*t*-butyl-4-methylphenol (Wako, Osaka, Japan) were added. The tube was mixed by vortexing for 1 min, followed by centrifugation at 12,500 × *g* for 5 min at 4°C. The supernatant (500 µl) was either stored at −80°C or analyzed immediately to detect lipid peroxidation products. Lipid peroxide contents were normalized to the protein concentration, which was measured by BCA protein assay.

### Analysis of lipid peroxides

To analyze lipid peroxides, the concentration of 7β-hydroxycholesterol (7β-OHCh), a cholesterol-derived peroxidation product, 4 isomers of hydroxyoctadecadienoic acid (HODE), which are linoleate-derived peroxidation products, and 3 isomers of hydroxyeicosatetraenoic acid (HETE) and 8-iso-prostaglandin F_2α_ (8-iso-PGF_2α_), which are arachidonate-derived peroxidation products, were measured by liquid chromatography-tandem mass spectrometry (LC-MS/MS) (HODEs, HETEs, and 8-iso-PGF_2α_) or gas chromatography-mass spectrometry (7β-OHCh) as previously described.^(25–27)^ 13-Hydroxy-9(*E*), 11(*E*)-octadecadienoic acid [13-(*E*,*E*)-HODE], 9-hydroxy-10(*E*), 12(*E*)-octadecadienoic acid [9-(*E*,*E*)-HODE], 7β-OHCh, and 8-iso-PGF_2α_ are specific products of radical-mediated oxidation. 13-Hydroxy-9(*Z*),11(*E*)-octadecadienoic acid [13-(*Z*,*E*)-HODE], and 5-, 12-, and 15-HETE are generated by both radical-mediated oxidation and enzymatic oxidation. Total HODE (tHODE) indicates the sum of the 4 isomers of HODEs.

### Knockdown of 5-lipoxygenase in MDCK cells

For RNA interference, sequences were produced as described by Lisovyy *et al.*^(28)^ A 5-lipoxygenase (5-LOX)-specific short interfering RNA (siRNA) (sense 5'-GCAAGAAGCUACCCGAGAAUU-3', and antisense 5'-UUCUCGGGUAGCUUCUUGCUU-3') was prepared from corresponding oligonucleotides provided by Dharmacon (Lafayette, CO) according to the manufacturer’s protocol. MDCK cells were transfected with siRNA (5 nM final concentration) using lipofectamine RNAiMAX (Invitrogen, Carlsbad, CA) according to the manufacturer’s instructions. At 24 h after siRNA transfection, the cells were placed in fresh medium. At 48 h after siRNA transfection, viruses (MOI 0.001) were infected for 1 h, and the inoculum was removed, washed once with serum-free MEM, and replaced with FBS-free DMEM supplemented with or without 275 µM daidzein.

### Western blot analysis of 5-LOX protein

Whole cell lysates of MDCK cells were prepared by homogenization in RIPA buffer. The whole cell lysate of MDCK cells was subjected to sodium dodecyl sulfate-polyacrylamide gel electrophoresis and 5-LOX and actin were analyzed by immunoblotting using an anti-5-LOX monoclonal antibody (ab169755, dilution 1/1,000; Abcam, Cambridge, UK) and anti-actin antibody (MAB1501R, dilution 1/2,000; Chemicon International, Temecula, CA).

### Evaluation of LOX activities in MDCK cell lysates

 The cells for analysis of 5-LOX activity were prepared by culturing non-infected monolayer sheets of MDCK cells in 10-cm dishes supplemented with or without 275 µM daidzein (mock-treated cells: 3 dishes and daidzein-treated cells: 3 dishes). After 24 h, the dishes were washed twice with PBS. The cells were harvested into 1.5-ml tubes containing 500 µl of 2% bovine serum albumin containing DMEM using a cell-scraper. The cells were homogenized and then evenly divided into six tubes. Arachidonic acid (AA) was added to each tube to a final concentration of 120 µM and incubated for 180 min at 37°C with gentle mixing with a microtube mixer. After 180 min of incubation, 4 volumes of methanol containing 100 µM 2,6-di-*t*-butyl-4-methylphenol (Wako) was added. The tubes were mixed by vortexing for 1 min, and then centrifuged at 12,500 × *g* for 5 min at 4°C. The supernatant (500 µl) was either stored at −80°C or used to detect 5-, 12-, and 15-HETE and AA. HETEs and AA were measured as described above for lipid peroxide analysis. The content of HETEs and AA was normalized to the protein concentration, which was measured by BCA protein assay.

### Cytotoxicity analysis

The WST-8 assay [using Cell Counting Kit-8 (Dojindo Laboratories, Kumamoto, Japan)] is a modified MTT assay that measures the mitochondrial reduction capacity and can quantify cell viability.^(29)^ After treatment with daidzein or 5-hydroperoxyeicosatetraenoic acid (5-HpETE), the cells in 96-well plates were incubated with 10 µl of Cell Counting Kit-8 solution {containing with 4-[3-(2-methoxy-4-nitrophenyl)-2-(4-nitrophenyl)-2H-5-tetrazolio]-1,3-benzene disulfonate sodium salt} in medium for 1 h at 37°C. Absorbance was measured photometrically at 450 nm. Cell viabilities were expressed as a percentage of the absorbance measured in non-treated cells.

### Statistical analysis

Statistical analyses were performed using unpaired *t* test and analysis of variance with Tukey-Kramer test using SPSS ver. 21.0 software (SPSS, Inc., Chicago, IL). A *p* value of less than 0.05 was considered to indicate significance.

## Results

### Effect of virus replication and lipid peroxide production in MDCK cells by daidzein addition

**Fig. 1:**
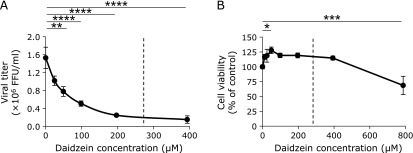
Effect of daidzein on multiplication of influenza virus and on proliferation of MDCK cells. MDCK cells were inoculated with influenza A/PR/8/34 virus at a MOI of 0.001. (A) Concentration-dependent inhibitory effect of Daidzein on virus multiplication. Viral titers were determined at 24 h post-infection by focus-forming assays. (B) Cytotoxicity of daidzein. The cell viability of MDCK cells was determined at 24 h post-addition of daidzein by WST-8 assay. The dotted lines indicate a daidzein concentration of 275 µM. Data are presented as mean ± SD (*n* = 3). Data are representative of three independent experiments. *p<0.01; **p<0.05; ***p<0.0025; ****p<0.001.

The titer of influenza virus in the culture medium decreased in a dose-dependent manner following addition of daidzein (Fig. 1A). Daidzein inhibited influenza virus multiplication at an IC_50_ of 51.2 µM (Fig. 1A). The effect of daidzein addition on the cell viability of MDCK cells was analyzed, however, it was found that cell proliferation was not affected by up to 400 µM of daidzein (Fig. 1B). To clarify the mechanism of antiviral activity of daidzein, the following experiments were performed with the addition of a daidzein concentration fixed at 275 µM. This concentration of daidzein in the culture medium inhibited influenza virus multiplication by approximately 85% and did not exert toxicity on MDCK cells.

**Fig. 2:**
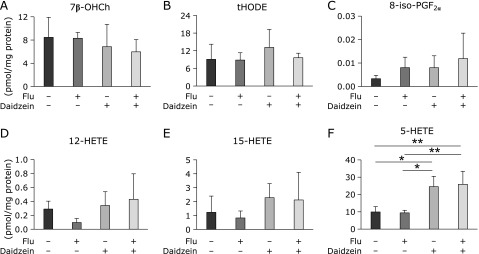
Effect of daidzein on production of lipid peroxide. MDCK cells were inoculated with influenza A/PR/8/34 virus at a MOI of 0.001. Lipid fraction was harvested from MDCK cells at 24 h post-infection. (A) 7β-OHCh, (B) tHODE, (C) 8-iso-PGF_2α_, (D) 12-HETE, (E) 15-HETE, and (F) 5-HETE in cells. Data are presented as mean ± SD (*n* = 3). Data are representative of three independent experiments. *p<0.01, **p<0.005.

The contents of lipid peroxide in the cells are shown in Fig. 2A–F. Lipid peroxide was not elevated in virus-infected cells (Fig. 2A–F). Although daidzein is known as an antioxidant, lipid peroxide was not decreased in daidzein-treated cells (Fig. 2A–F). These results indicate that daidzein did not exert antiviral activity through its antioxidant function under the conditions of this experiment. In contrast, 5-HETE content in the cells significantly increased in daidzein-treated cells both with and without virus infection (Fig. 2F), although the contents of 7β-OHCh (Fig. 2A), tHODE (Fig. 2B), 8-iso-PGF_2α_ (Fig. 2C), 12-HETE (Fig. 2D), and 15-HETE (Fig. 2E) were not significantly increased in daidzein-treated cells.

**Fig. 3:**
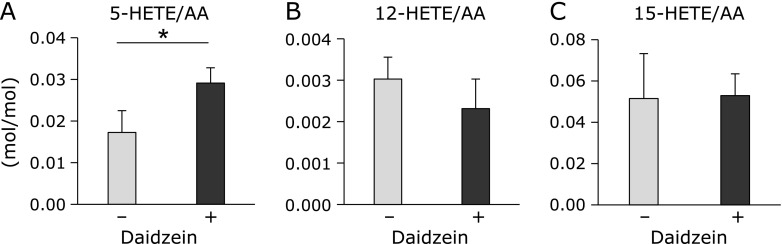
Effect of daidzein on lipoxygenase activity in MDCK cell lysate. The cell lysates were obtained from MDCK cells treated for 24 h with or without daidzein. AA was added to cell lysates to a final concentration at 120 µM and incubated for 180 min at 37°C with gentle mixing. After 180 min incubation, lipids were extracted and 5-, 12-, and 15-HETE and AA were measured by LC-MS/MS. The 5-, 12-, and 15-LOX activities in MDCK cell lysates were evaluated as the ratio of each HETE to AA. (A) 5-LOX activity was evaluated as 5-HETE/AA, (B) 12-LOX activity was evaluated as 12-HETE/AA, and (C) 15-LOX activity was evaluated as 15-HETE/AA. Data are presented as mean ± SD (*n* = 3). Data are representative of three independent experiments. *p<0.05.

As daidzein addition specifically induced a significant 5-HETE production in MDCK cells, we hypothesized that daidzein activated 5-lipoxygenase (5-LOX), which converts arachidonic acid to 5-HpETE, a precursor of 5-HETE. We analyzed the 5-, 12-, and 15-LOX activities in MDCK cell lysates. The cell lysates were obtained from mock-treated MDCK cells or those treated with daidzein and incubated with 120 µM arachidonic acid (AA). After 180 min incubation, 5-, 12-, and 15-HETE concentrations in the reactants were analyzed by LC-MS/MS. As shown in Fig. 3A, 5-HETE production in the daidzein-treated cell lysate was significantly higher than that in the mock-treated cell lysate. In contrast, daidzein treatment did not activate 12-HETE (Fig. 3B) and 15-HETE (Fig. 3C) production. These data indicate that daidzein enhances the enzymatic activity of 5-LOX.

### Effect of zileuton on influenza virus replication in daidzein added virus-infected cells

**Fig. 4:**
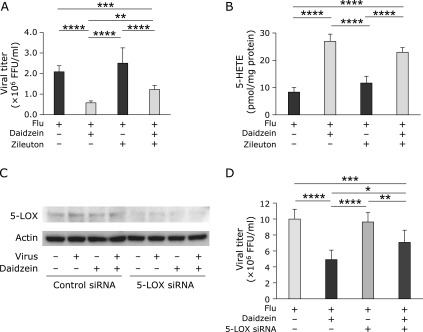
Effect of 5-LOX inhibitor, zileuton, and 5-LOX siRNA transfection on multiplication of influenza virus and production of 5-HETE. MDCK cells were inoculated with influenza A/PR/8/34 virus at a MOI of 0.001. Viral titers were determined at 24 h post-infection by focus-forming assays. (A) Effect of zileuton on the titer of influenza virus. (B) Effect of zileuton on 5-HETE production. (C) Effect of 5-LOX siRNA on 5-LOX expression in whole cell lysate of MDCK cells treated with or without daidzein and with or without influenza virus infection. (D) Effect of 5-LOX siRNA on daidzein-induced inhibition of virus multiplication. Data are presented as mean ± SD. Data are representative of three independent experiments. *p<0.05, **p<0.025, ***p<0.005, ****p<0.001.

To confirm that 5-LOX is involved in the daidzein-induced antiviral activity, we examined the effect of inhibitors of 5-LOX on antiviral activity. The antiviral activity of daidzein was significantly reduced following treatment with zileuton, a selective direct inhibitor of 5-LOX (Fig. 4A). Daidzein-induced inhibition of virus replication was decreased in the presence of zileuton from 72% [flu: flu + daidzein, inhibition rate = 100 – 100 × 0.58 × 10^6^/2.1 × 10^6^ (%)] to 51% [flu + zileuton: flu + daidzein + zileuton, inhibition rate = 100 – 100 × 1.2 × 10^6^/2.5 × 10^6^ (%)] (Fig. 4A). The virus titer was increased by zileuton to 212% [flu + daidzein: flu + daidzein + zileuton, activation rate = 100 × 1.2 × 10^6^/0.58 × 10^6^ (%)] (Fig. 4A). Zileuton tended to reduce daidzein-induced 5-HETE production, but the reduction was not significant (Fig. 4B).

In contrast, when MK-886 was used to indirectly inhibit 5-LOX, daidzein-induced antiviral activity was not inhibited (Supplemental Fig. 1A*). Additionally, MK-886 did not affect 5-HETE production in daidzein-treated cells (Supplemental Fig. 1B). This suggests that MK-886 does not inhibit 5-LOX activity in MDCK cells.

### Effect of 5-LOX knockdown on antiviral activity of daidzein

We hypothesized that daidzein affected 5-LOX-mediated enzymatic oxidation of AA, as zileuton inhibited daidzein-induced antiviral activity. We attempted to knockdown endogenous 5-LOX by transfection with a siRNA against canine-5-LOX. Based on the immunoblotting results (Fig. 4C), 5-LOX expression levels in MDCK cells were suppressed. We then measured the influenza virus titer in the culture medium. In control siRNA-transfected MDCK cells, daidzein inhibited virus replication by approximately 50.8% [flu: flu + daidzein, inhibition rate = 100 – 100 × 4.91 × 10^6^/9.99 × 10^6^ (%)] (Fig. 4D). In contrast, following knockdown of 5-LOX, daidzein reduced virus replication to 26.5% [flu: flu + daidzein, inhibition rate = 100 – 100 × 7.07 × 10^6^/9.62 × 10^6^ (%)] (Fig. 4D). The difference in the viral titer between daidzein-treated control siRNA MDCK cells (4.91 ± 1.18 × 10^6^ FFU/ml) and daidzein-treated 5-LOX knockdown MDCK cells (7.07 ± 1.53 × 10^6^ FFU/ml) was significant (*p*<0.05). These results indicate that 5-LOX is involved in the inhibitory effect of daidzein on virus replication.

### Effect of 5-HpETE administration on viral replication in virus-infected MDCK cells

**Fig. 5:**
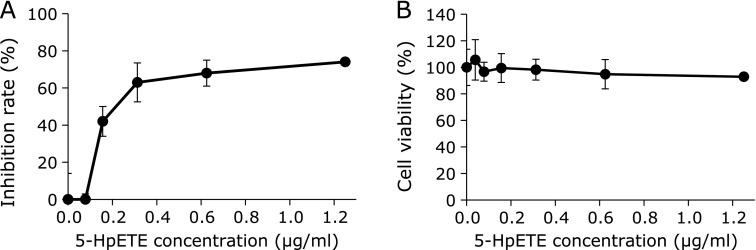
Effect of 5-HpETE on multiplication of influenza virus. MDCK cells were inoculated with influenza A/PR/8/34 virus at a MOI of 0.001. (A) Concentration-dependent inhibitory effect of 5-HpETE on virus multiplication. The inhibition rate of the virus titer was determined at 24 h post-infection by focus-forming assays. (B) Cytotoxicity of 5-HpETE. The survival rate of MDCK cells was determined at 24 h post-infection by WST-8 assay. Data are presented as mean ± SD (*n* = 3). Data are representative of three independent experiments.

Because 5-LOX was found to be involved in daidzein-induced inhibition of virus replication, we confirmed whether 5-LOX product inhibits virus proliferation. Administration of 5-HpETE, a precursor of 5-HETE and 5-LOX primary product derived from AA, inhibited virus proliferation in a concentration-dependent manner (Fig. 5A) with an IC_50_ of 204.0 ng/ml (606.3 nM). 5-HpETE showed nearly no cytotoxicity at concentrations up to 1,250 ng/ml (Fig. 5B). These results indicate that 5-HpETE or its metabolite regulate virus replication.

## Discussion

The present study clarified the mechanism of daidzein-induced inhibition of influenza virus replication. Daidzein showed no antioxidant activity under our experimental conditions (Fig. 2A–F); however, it enhanced 5-HETE production (Fig. 2F). Based on the effects of zileuton, a direct inhibitor of 5-LOX (Fig. 4A and B), and knockdown of 5-LOX (Fig. 4D), we observed that 5-LOX is involved in daidzein-induced 5-HETE production and daidzein-induced anti-influenza activity. Additionally, 5-HpETE, the primary product of 5-LOX, inhibited influenza virus replication (Fig. 5A).

This is the first study to demonstrate that daidzein activates 5-LOX and regulates viral replication. No previous studies have reported exogenous compounds that can increase 5-LOX activity, although 5-lipoxygenase-activating protein (FLAP) is well known as an endogenous 5-LOX activator.^(30)^ The food component daidzein enhances 5-LOX activity and can be used to prevent and treat influenza. Liu *et al.*^(31)^ reported the effect of soy isoflavone administration to non-alcoholic fatty liver disease model rat. And they showed that soy isoflavones affected the expression of sterol regulatory element binding protein (SREBP)-1c and peroxisome proliferator-activated receptor (PPAR) α.^(31)^ There may be potential signal transduction pathways to enhance 5-LOX activity by daidzein.

It has been reported that oxidation products derived from omega-3 and omega-6 polyunsaturated fatty acids (PUFAs) have various functions such as causing and resolving inflammation and are involved in signal transduction as lipid mediators.^(32,33)^ Morita *et al.*^(34)^ reported that the omega-3 PUFA-derived lipid mediator protectin D1 (PD1) attenuates influenza virus replication. They performed a screen of PUFA-derived lipid metabolites in human lung epithelial (A549) cells infected with influenza A virus (PR8 virus) by using bioactive lipid libraries containing prostaglandins, resolvins, protectins, and other PUFA-derived metabolites.^(34)^ They evaluated the antiviral effect by influenza virus nucleoprotein mRNA expression at 8 h after infection and found that 12-HETE, 15-HETE, 17-hydroxy-4*Z*,7*Z*,10*Z*,13*Z*,15*E*,19*Z*-docosahexaenoic acid, or PD1 treatment of PR8-virus-infected A549 cells inhibited nucleoprotein mRNA expression.^(34)^ Results of their report indicate that there are several PUFA-derived metabolites, which have anti-influenza activity, produced by lipid-metabolizing enzymes such as lipoxygenase. We demonstrated the antiviral activity of 5-HpETE in the present study (Fig. 5A); however, the mechanism remains unclear. 5-HpETE is further metabolized to leukotrienes by leukotriene-converting enzymes and leukotrienes exert physiological functions in the pathophysiology of asthma and inflammation.^(35)^ Additionally, there is another 5-LOX associated arachidonate-derived metabolite: lipoxin A4.^(36)^ It is possible that daidzein-induced enzymatic activation of 5-LOX produced 5-LOX-associated PUFA-derived metabolites other than 5-HpETE and that these metabolites exert antiviral activity. Further studies are needed to determine whether 5-HpETE or another 5-LOX-associated PUFA-derived metabolite exerts antiviral functions and how these lipid metabolites suppress viral replication.

Zileuton, a direct inhibitor of 5-LOX, significantly reduced the antiviral activity of daidzein (Fig. 4A). However, the reduction effect of zileuton on daidzein-induced 5-HETE production was not significant (Fig. 4B). As shown in Fig. 5A, the inhibition rate of viral replication was elevated sharply at low concentrations of 5-HpETE (<200 ng/ml). This indicates that even a slight reduction in 5-HETE by zileuton addition can affect viral replication. Additionally, 5-HETE is produced in cells not only by 5-LOX-mediated enzymatic oxidation, but also by oxidation by ROS. Although we did not determine the proportion of 5-HETE generated by ROS relative to the total 5-HETE level, zileuton did not effectively suppress 5-HETE production because zileuton did not suppress ROS-mediated 5-HETE generation. This may be because zileuton could not significantly reduce 5-HETE generation without daidzein administration (Fig. 4B). As shown in Supplemental Fig. 1, MK-886, an inhibitor of 5-LOX, did not suppress 5-HETE production. MK-886 inhibits FLAP, which is thought to facilitate the transfer of phospholipid-derived AA to 5-LOX.^(30)^ It is thought that MK-886 cannot inactivate FLAP in MDCK cells.

The addition of daidzein showed anti-influenza activity with an IC_50_ of 51.2 µM (Fig. 1A). King *et al.*^(37)^ reported the pharmacokinetics of the soy isoflavones daidzein and genistein in humans. Subjects consumed single soybean flour-based meal (0.84 g flour/kg body weight) and plasma concentration of isoflavones were analyzed throughout the 35-h post-meal period. This soybean flour-based meal provided 2.7 µmol/kg body weight of daidzein. Daidzein concentration in plasma reached maximum values of 3.14 ± 0.36 µmol/L.^(37)^ A subject weighting 60 kg would have consumed 41.2 mg (162 µmol) of daidzein. Based on the results of this report, in order to increase the plasma concentration of daidzein to 51.2 µM, the IC_50_ for anti-influenza activity, it is estimated that 667.4 mg of daidzein must be consumed. On the other hand, Cheng *et al.*^(38)^ reported the effect of a high dose of isoflavones (300 mg/day) on coagulation function in postmenopausal women. After 1-year treatment with a high dose of isoflavones, the changes in liver function, hematological parameters, and coagulation test were not different from those of the control subjects.^(38)^ The dose of isoflavones administered in this clinical study corresponds to less than half of the estimated dose of daidzein that can exert anti-influenza activity. The duration of daidzein administration for the treatment of influenza infection is thought to be 5 days. Further research is necessary to examine the safety of short-term high dose administration of daidzein.

The inhibitory mechanism of daidzein against influenza replication does not involve the inhibition of viral enzymes, such as neuraminidase and cap-dependent endonuclease, which are encoded by the influenza virus genome, but rather involves enzyme activation in infected host cells. The results of the present study provide a foundation for developing anti-influenza treatments with a novel mode of action, as well as for developing preventive strategies against influenza infection using food ingredients.

## Author Contributions

YH, RS, MS, NI, and RM performed experiments and analyzed the data; AO provided new tools and reagents; YI conceived and supervised the study; YI designed experiments and wrote the manuscript.

## Abbreviations

7β-OHCh: 7β-hydroxycholesterol
FFRA: focus-forming reduction assay
FFU: focus-forming unit
FLAP: 5-lipoxygenase-activating protein
HETE: hydroxyeicosatetraenoic acid
HODE: hydroxyoctadecadienoic acid
HpETE: hydroperoxyeicosatetraenoic acid
8-iso-PGF2_2α_: 8-iso-prostaglandin F_2α_
LC-MS/MS: liquid chromatography-tandem mass spectrometry
5-LOX: 5-lipoxygenase
MDCK: Madin-Darby canine kidney
MOI: multiplicity of infection
PD1: protectin D1
PUFA: polyunsaturated fatty acid
ROS: reactive oxygen species
siRNA: short interfering RNA
tHODE: total hydroxyoctadecadienoic acid
