# Supplementary material for: Induction of a 5-lipoxygenase product by daidzein is involved in the regulation of influenza virus replication
Source: J Clin Biochem Nutr. 2020 Jan 1;66(1):36–42. doi: 10.3164/jcbn.19-70 (PMC6983437; doi:10.3164/jcbn.19-70)
Supplement: Supplemental Figure 1 [file jcbn19-70sf01.pdf]

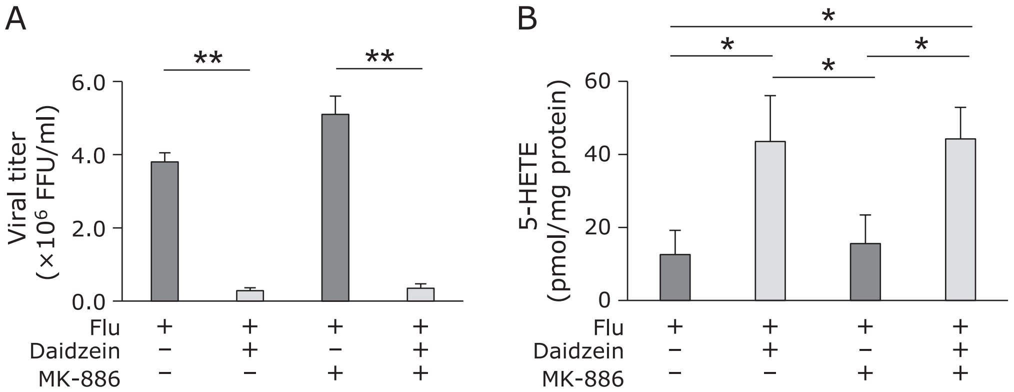

Supplemental Fig. 1. Effect of FLAP inhibitor, MK-886, on multiplication of influenza virus and production of 5-HETE. MDCK cells were inoculated with influenza A/PR/8/34 virus at a MOI of 0.001. (A) Effect of MK-886 on titer of influenza virus. Viral titers were determined at 24 h post-infection by focus-forming assays. (B) Effect of MK-886 on 5-HETE production. Data are presented as mean ± SD (n = 3). Data are representative of three independent experiments. *p<0.005, **p<0.001.
